# Improve the quality of counseling for HIV in the district of Guédiawaye

**DOI:** 10.1186/1742-4690-9-S1-P86

**Published:** 2012-05-25

**Authors:** Sakho D Maty, Seck Karim, Ouattara Baly, Gaye Alioune, Sylla Bintou

**Affiliations:** 1 Médecin Spécialiste du Vih at District Sanitaire de Guédiawaye Synergie pour l'Enfance, Dakar, Senegal

## Objectives

HIV counseling and testing for several years working in the district of Guédiawaye. A situation analysis has revealed shortcomings in the organization of the service given the many other activities of the health center. This is why the medical team decided to use the collaborative model of quality improvement that is based on the implementation of a package of changes and the measurement of indicators of improvement.

## Methodology

It consisted of a situational analysis of the Board Search, the development of process maps and identify targets for improvement. Improvement targets were selected to make the results of all HIV tests to clients within two hours, to provide the reference of all patients positive for medical care. The changes have been to extend the opening hours of the laboratory, to assign a specific staff for testing and to ensure continuous availability of a pool of consultants. Better communication was established and the customer's circuit has been reduced.

## Results

From January to December 2010, the proportion of tests performed in two hours has risen by an average of 35% to 99%, also the proportion of patients actually referred for medical care rose from an average of 33% to 97%.

## Conclusions and recommendations

The process of improving not only affects the indicators of improvement but the effects on teamwork and a consideration of the basic needs of patients.

